# Cognitive Impairment in Older Adults With Concurrent Hearing and Vision Impairment: A Systematic Scoping Review Protocol

**DOI:** 10.3389/fpsyt.2021.661560

**Published:** 2021-07-19

**Authors:** Atul Jaiswal, Natalina Martiniello, Peter Holzhey, Gabrielle Aubin, Shirley Dumassais, Stephanie Huang, Geneviève Major, Roxane Mirmiran, Fatima Tangkhpanya, Norman Robert Boie, Walter Wittich

**Affiliations:** ^1^School of Optometry, Université de Montréal, Montreal, QC, Canada; ^2^Centre for Interdisciplinary Research in Rehabilitation of Greater Montreal/Institut Nazareth et Louis-Braille du Centre Intégré de Santé et de Services Sociaux de la Montérégie-Centre, Longueuil, QC, Canada; ^3^Interdisciplinary School of Health Sciences, Faculty of Health Sciences, University of Ottawa, Ottawa, ON, Canada; ^4^Centre for Interdisciplinary Research in Rehabilitation of Greater Montreal/Centre de Réadaptation Lethbridge-Layton-Mackay du Centre Intégré Universitaire de Santé et de Services Sociaux du Centre-Ouest-de-l'Île-de-Montréal, Montreal, QC, Canada

**Keywords:** dual sensory impairment, prevalence, incidence, risk factors, screening tools, older adults, cognitive impairment, review

## Abstract

**Introduction:** As the prevalence of age-related sensory impairment increases, more evidence emerges on the association between uni-sensory and cognitive impairment (CI) in older adults. However, the link between CI and concurrent hearing and vision impairment (referred to as dual sensory impairment/DSI) is not well-understood, and this combined effect may be additive or multiplicative. Moreover, the existing evidence on CI in older adults with DSI is scattered and limited. Through this systematic scoping review, we aim to map existing evidence on CI in older adults with DSI, and to summarize what is known about the prevalence, incidence and risk factors of CI, and tools used to screen or assess CI in older adults with DSI.

**Methods and Analysis:** We will use the Joanna Briggs Institute framework to perform the review. Eleven databases [MEDLINE, CINAHL/EBSCO, EMBASE, Mednar, WorldWideScience, PsycEXTRA, OAIster, OpenGrey (SIGLE), Global Health, PsycINFO, and Web of Science] and clinical trial registries (ISRCTN Registry, WHO ICTRP, and ClinicalTrials.gov) will be searched. Study selection will be completed using Covidence, and data will be extracted using an *a priori* data extraction tool. To be included, studies had to be peer-reviewed, had older adults with DSI as the focal population, and are related to CI. Data will be presented using a narrative summary with emphasis on implications for future research and practice.

**Discussion:** Reliable cognitive screening is of the utmost importance for prevention and treatment of CI within DSI population. The study findings will have significant implications for health services delivery and policy research. The summarized findings on the prevalence, incidence, associated risk factors, and CI screening and assessment tools will inform geriatric care. The review will also document knowledge gaps on CI in the DSI population and identify areas of interest for future studies.

**Ethics and Dissemination:** The scoping study, being a review of existing documents, does not require ethics approval. The findings will be disseminated with relevant stakeholders using knowledge translation activities such as scientific presentations and publications. We intend to use the findings to conduct a Delphi study to evaluate which CI tools are suitable for older population with DSI.

## Introduction

Although vision and hearing impairments commonly occur separately, the prevalence of individuals with concurrent vision and hearing impairment is increasing as the global population continues to age (1). Dual sensory impairment (DSI) refers to the presence of a concurrent vision and hearing impairment in the same individual, independently of whether both impairments are congenital or acquired (1–3). The heterogeneous nature of this population renders it difficult to define. Definitions vary based on age and order of onset, as well as the respective severity of vision and hearing impairment (2, 3). In the medical domain, DSI is defined based on objective measures of hearing and vision (such as visual acuity or pure tone average). Educational and rehabilitation definitions place a greater emphasis on functional impacts on activities of daily living (communication, information access and independent travel) (2–4). A combination of age-related conditions, such as age-related macular degeneration, cataract, glaucoma, diabetic retinopathy, as well as age-related central and peripheral hearing loss, and age-related central auditory processing disorder (CAPD) could lead to combined sensory impairment among older adults (2, 4–9). Older adults with DSI can be categorized into three distinct groups: (1) those who live with visual and hearing impairments for all or most of their life; (2) those who live with a single sensory impairment and later in life acquire a second one; and (3) those who acquire both sensory impairments in old age (10). Given that the majority of individuals with DSI who seek rehabilitation services are over the age of 65, and that this group is expected to grow, there is an increasing need for research focused on the unique implications of DSI in older adulthood (5).

Regardless of the definition, individuals with DSI experience varying degrees of functional limitations, which impede communication, information access, independent travel and other activities of daily living (4, 6, 11). These limitations contribute to reduced social participation and pose a higher risk of social isolation, depression and cognitive impairment (CI) (6, 11, 12). Evidence suggests a strong association between sensory impairment and CI (13). The communication barriers experienced by individuals with DSI may limit functioning and social participation and therefore increase the likelihood of developing CI (14). Proper identification of CI in this population is vital for designing effective rehabilitation interventions and support, including clinical-decisions about which assistive devices and techniques will be most appropriate for specific client needs of those living with DSI (15–17). Moreover, less is known about the prevalence, incidence, and risk factors associated with CI in older adults with DSI. This epidemiological information is crucial for large scale population-based studies in future to inform effective health service delivery and policy action.

Despite the importance of accurately understanding the cognitive abilities of individuals with sensory impairments, current cognitive screening tools rely on the integrity of vision and hearing in patients. Two of the most commonly used tools for cognitive screening, the Montreal Cognitive Assessment (MoCA) and the Mini-Mental State Examination (MMSE), both have vision- and hearing-dependent items (18). Therefore, if visual and hearing impairments are not considered during test administration, the cognitive abilities of individuals with sensory impairment(s) are likely to be underestimated (18). For example, individuals with visual impairments will score lower than sighted control participants on cognitive tests where items are vision dependent; however, when vision-independent items are added to the cognitive testing, there is no significant difference (19). In order to adequately screen for CI in individuals with a vision or hearing impairment, tests need to only minimally rely on sensory function. To address some of these concerns, the MoCA has been adapted for individuals with a visual impairment (MoCA-Blind) (20). This adapted version excludes the visual items of the MoCA and puts forth a new cut-off value to indicate a passing score. The MoCA has also been adapted for individuals with severe hearing impairments (HI-MoCA) (15). Rather than having the experimenter read the instructions aloud, this information is available in written format on a timed PowerPoint presentation.

These adapted versions of the MoCA adequately screen for risk of mild CI in individuals with single sensory impairment because the administration compensates by relying on the healthy sensory modality (vision or hearing, respectively) (11, 20). However, for individuals with DSI, reliance on vision or hearing is not possible because both are impaired. Recognizing the limitation of existing assessments, some tests have been developed to assess cognition in individuals with DSI, including the Tactile Test Battery and the Tactile Short-term Memory Task (21, 22). Both these studies provide promising adapted cognitive screening tools for individuals with DSI. However, both focus on specific cognitive functions such as learning, memory, naming, visuospatial perception, and processing speed (21) and short-term memory (22). Disorders that effect other executive functions, such as in frontotemporal dementia, may be overlooked (23). Therefore, there is a need for the development of adapted tests that assess a larger number of cognitive functions in individuals with DSI.

Parallel efforts have explored the use of tools that are suitable for screening vision and hearing function in persons with dementia (24). This work has resulted in recommendations of which tools for vision (25) and hearing (26) may be most suitable for use by nurses in long-term care settings (27) where the prevalence of DSI and CI is disproportionally high (28). Our efforts reverse this approach by exploring which measures of CI have been adapted for or used with older persons with DSI.

There is a need for research, rehabilitation and the implementation of appropriate interventions which help to maintain the cognitive well-being of individuals with DSI. Interventions informed by accurate information about CI will facilitate the use of appropriate techniques and better optimize overall health (13). Research also suggests that caregivers of individuals with DSI experience higher levels of anxiety and stress compared to caregivers of individuals with CI alone. Consequently, the use of interventions that maximize independence also carry important implications for caregivers of individuals with DSI (13).

To avoid duplication, we conducted a preliminary search in PROSPERO, MEDLINE, Cochrane Database of Systematic Reviews, and the Joanna Briggs Institute (JBI) *Evidence synthesis*. There is currently no comprehensive review focusing on CI in older adults with DSI. The objective of this scoping review is to map existing evidence on CI in older adults with DSI, and to summarize what is known about the prevalence, incidence and risk factors of CI in this population. Additionally, we will identify and present available tools to screen or assess CI that have been used in studies with older adults with DSI, and whether and how their administration was adapted to this population.

## Review Question(s)

1. What evidence exists on the prevalence, incidence, and risk factors associated with CI in older adults with DSI?
2. Which tools have been used in studies with older adults with DSI to screen or assess their CI?

## Inclusion Criteria

### Participants

This review will include studies that focus on older adults with DSI. Given that age classification of older adults varies depending on the setting and the country, all studies that recruited older adults based on their own defined criteria for age cut-off will be included in this review. We use 60 years as the age-cut-off to define older adults. Studies on only children (0–18 years) or working-age adults (18–59 years) will not be included. Based on the Nordic definition, DSI refers to a combined vision and hearing loss in the same individual that results with functional limitations in relation to an individual's activities and social participation in the society (29). All combinations of DSI (e.g., congenital or acquired, pre-lingual or post-lingual, mild to a total absence of vision, and hearing loss) will be included, regardless of terminology (e.g., dual sensory impairment, deafblindness, combined vision, and hearing impairment). Studies with older adults with pre-existing cognitive impairment/dementia, in addition to DSI, will be included. We will be including characteristics of the study population such as race, gender, and geographic location. Studies focusing specifically on physiology or genetic characteristics will be excluded.

### Concept

This review will consider studies that explore the concept of CI in relation to DSI. From a psychometric approach, “cognitive impairment” refers to an impairment that can range from mild to severe of one or more cognitive domains, such as memory, attention, or executive functions (30). We will include cognitive measures that cover global or specific cognitive functions. For example, both the MMSE and MOCA assess multiple cognitive domains; however, the MOCA incorporates more executive function tasks. Genetic and animal model studies in relation to this concept will be excluded.

### Context

This review will consider studies from across the globe that are related to CI in older adults with DSI.

### Types of Studies

This review will consider studies that (1) are original research studies of any study design, including randomized controlled trials, observational, descriptive, or cohort studies, case-control studies, cross-sectional studies, qualitative studies, and mixed methods studies, (2) include studies where participants were 60 years old or older, (3) focus on participants with DSI (defined subjectively through self-report or objectively using standardized screening or assessment tests for hearing and vision impairment). Studies published in any language will be included where English translation is available. Editorials, conference publications, thesis/dissertations, books or letters will be excluded. There is no date restriction applied, and databases are searched since inception.

## Methods

The protocol was developed using the Preferred Reporting Items for Systematic Review and Meta-Analysis Protocols (PRISMA-P) statement (31) (Additional file 1: PRISMA-P checklist). The proposed systematic scoping review will be conducted in accordance with the Joanna Briggs Institute methodology for systematic scoping reviews (32). We will use the PRISMA Extension for Scoping Reviews (PRISMA-ScR) for reporting of the systematic scoping review (33).

### Search Strategy

A comprehensive literature search of scientific databases will be performed in accordance with the Preferred Reporting Items for Systematic Reviews (PRISMA) (31). Search terms will be determined based on the key concepts arising from the review objectives. Following the recommendations of JBI, a three-step search strategy will be developed in collaboration with a health science librarian. The first step is an initial limited search in MEDLINE to identify relevant keywords and search terms used in titles and abstracts published concerning the topic. This step is followed by an analysis of the key words contained in the title and abstract of retrieved papers, and of the index terms used to describe the articles. Then, those keywords will be expanded and tailored in order to fit with the terms found in the selected databases, using synonyms, alternate terms or different spellings. The two main concepts to explore will be DSI and CI. The different search strings are presented in Appendix 1. The reference list of selected relevant studies will be screened for additional studies. The purpose of selecting eleven scientific databases is to maximize the comprehensiveness of the scoping process and to avoid missing key articles.

#### Information Sources

The scientific databases to be searched include MEDLINE, CINAHL/EBSCO, EMBASE, Mednar, WorldWideScience, PsycEXTRA, OAIster, OpenGrey (SIGLE), Global Health, PsycINFO, and Web of Science. To supplement our electronic search, we will search clinical trials registries such as www.ClinicalTrials.gov, BioMed Central ISRCTN registry, and http://www.who.int/trialsearch/.

### Study Selection

Following the search, all identified citations will be exported to EndNote X9 (Clarivate Analytics, PA, USA). Titles and abstracts will then be imported to the Covidence review software (Veritas Health Innovation, Melbourne, Australia) for screening. Duplicates will automatically be removed by the software. Each title and abstract will be screened by two independent reviewers using the inclusion criteria (see Table 1). At the title/abstract level, studies mentioning vision, hearing, and cognition will be included. All full texts of eligible studies will be imported to Covidence. Each article will then be screened by two independent reviewers. Studies not explicitly dealing with DSI will be excluded during the full-text review. All conflicts that arise between the reviewers at each stage of the study selection process will be resolved by a senior reviewer. The study selection process will be presented as a PRISMA flowchart generated by Covidence (31).

**Table 1:** Inclusion and exclusion criteria.

Included when:	Excluded when:
- Vision and hearing are measured or considered, and cognitive function/impairment is measured or considered. - Study participants are 60 years old or older. - Studies from anywhere, in any language, from any publication year.	- Only vision or hearing are considered, and cognitive impairment is not is measured or considered. - Only children (0–18 years) or working-age adults (18–59 years) are included in the study. - Dual sensory impairment of other sense (gustatory, smell etc.). - Animal model or genetics studies.

### Data Extraction

After the full-text review, eligible data will be extracted from studies included in the review by a team of independent reviewers using a pre-designed Microsoft Excel data extraction spreadsheet (Appendix 2). If needed, changes to the extraction chart will be made during the process to include potential information pertinent to the review objective. Any disagreements between reviewers on article eligibility will be resolved by a third, senior reviewer.

We will extract the following relevant data from the selected articles: author(s), year of publication, study origin (where the source was published or conducted), aims/purpose, population and sample, methodology, outcomes, and key findings relevant to scoping review question (32). The following data specific to DSI and CI will also be extracted: the prevalence, incidence, risk factors, screening or assessment tools, tools administration accommodations, as well as the terminology used in articles to refer to DSI and CI. In case of missing information or necessity of additional information, the authors of the respective article will be contacted. The descriptor “not available” will be used if any of the required information was missing from the source.

### Data Analysis and Presentation

After data charting will be complete, we will prepare a descriptive numerical summary (based on simple frequency counts of concepts, populations, characteristics or other fields of data) and perform a descriptive qualitative content analysis to present the key findings of the study (32). We will present data in the form of tables of study characteristics, participants' details, outcomes such as prevalence, incidence, associated risk factors, screening or assessment tools, and whether and how methods of administration of these tools were adapted. In addition, data will be presented using diagrammatic or tabular form and in a descriptive narrative summary format. We will also identify and present both advances and gaps in the evidence, with particular attention to implications for future research in the area of assessment of CI in older adults with DSI. We will report this study using the Preferred Reporting Items for Systematic reviews and Meta-Analyses extension for Scoping Reviews (PRISMA-ScR) Checklist (33).

## Discussion

This systematic scoping review aims to map the evidence on CI in older adults with DSI to better guide clinical assessment and interventions. The study findings will have significant implications for health services delivery and policy research. The epidemiological information on prevalence, incidence, and risk factors may inform large scale population-based studies in future. Reliable cognitive screening is of the utmost importance for prevention and treatment of CI within DSI population. The summarized findings on CI screening and assessment tools will inform geriatric care. The scoping review will also document important knowledge gaps on CI in DSI population and identify areas of interest for future studies.

## Ethics Statement

Ethical review and approval was not required for the study on human participants in accordance with the local legislation and institutional requirements. Written informed consent for participation was not required for this study in accordance with the national legislation and the institutional requirements.

## Author Contributions

AJ and WW contributed to the conception, design of the study, and edited the final manuscript for submission. AJ and PH developed the initial search with the help of librarian. All authors were involved in writing the first draft of the protocol manuscript, read, and approved the submitted version.

## Conflict of Interest

The authors declare that the research was conducted in the absence of any commercial or financial relationships that could be construed as a potential conflict of interest.
