# Supplementary material for: Cognitive Impairment in Older Adults With Concurrent Hearing and Vision Impairment: A Systematic Scoping Review Protocol
Source: Front Psychiatry. 2021 Jul 19;12:661560. doi: 10.3389/fpsyt.2021.661560 (PMC8326366; doi:10.3389/fpsyt.2021.661560)
Supplement: Supplementary file 1 [file Data_Sheet_1.docx]

**Appendix I: Search strategy**

MEDLINE

Search conducted on October 20, 2020

| **Search** | **Query** | **Records retrieved** |
| --- | --- | --- |
| #1 | ((((((audiologi*[Title/Abstract]) OR "auditory"[Title/Abstract]) OR "ear"[Title/Abstract]) OR "hearing"[Title/Abstract]) OR "listening"[Title/Abstract]) AND (((((((eye[Title/Abstract]) OR ocular[Title/Abstract]) OR oculo[Title/Abstract]) OR optic[Title/Abstract]) OR sight[Title/Abstract]) OR vision[Title/Abstract]) OR visual[Title/Abstract]) AND ((((((((((((((((((((((abnormalit*[Title/Abstract]) OR defect*[Title/Abstract]) OR deficit*[Title/Abstract]) OR "deficiency"[Title/Abstract]) OR "deficiencies"[Title/Abstract]) OR delay*[Title/Abstract]) OR "disability"[Title/Abstract]) OR "disabilities"[Title/Abstract]) OR "disabled"[Title/Abstract]) OR disease*[Title/Abstract]) OR disorder*[Title/Abstract]) OR disturbance*[Title/Abstract]) OR dysfunction*[Title/Abstract]) OR "handicapped"[Title/Abstract]) OR handicap*[Title/Abstract]) OR "impaired"[Title/Abstract]) OR impairment*[Title/Abstract]) OR "loss"[Title/Abstract]) OR "lost"[Title/Abstract]) OR patholog*[Title/Abstract]) OR problem*[Title/Abstract]) OR syndrome*[Title/Abstract])) OR ((((((((((deaf*[Title/Abstract]) OR "hearing loss"[Title/Abstract]) OR "hard of hearing"[Title/Abstract]) OR "auditory perceptual disorders"[MeSH Terms]) OR "deafness"[MeSH Terms]) OR "ear diseases"[MeSH Terms]) OR "hearing disorders"[MeSH Terms]) OR "hearing loss"[MeSH Terms]) OR "persons with hearing impairments"[MeSH Terms]) AND ((((((((((((((("blind"[Title/Abstract]) OR "blindness"[Title/Abstract]) OR "unsighted"[Title/Abstract]) OR low vis*[Title/Abstract]) NOT "double blind"[Title/Abstract]) NOT "single blind"[Title/Abstract]) OR "blindness"[MeSH Terms]) OR "eye abnormalities"[MeSH Terms]) OR "eye diseases"[MeSH Terms]) OR "vision disorders"[MeSH Terms]) OR "vision, low"[MeSH Terms]) OR "visually impaired persons"[MeSH Terms]) OR "retinal diseases"[MeSH Terms]) OR "retinal degeneration"[MeSH Terms]) OR "retinitis pigmentosa"[MeSH Terms])) OR ((((((((((((((((combined sensory[Title/Abstract]) OR dual sensory[Title/Abstract]) OR blind deaf) OR vision hearing) OR usher syndrome) OR alstrom syndrome) OR charge syndrome) OR refsum syndrome) OR norrie syndrome) OR mohr tranebjaerg syndrome) OR cockayne syndrome) OR stickler syndrome) OR wolfram syndrome) OR melas syndrome) OR branchio oculo facial syndrome) OR alport syndrome) | 46378 |
| #2 | ((((((((((((((((((((((("cognitive confusion"[Title/Abstract]) OR "cognitive damage"[Title/Abstract]) OR "cognitive damages"[Title/Abstract]) OR ("cognitive decline"[Title/Abstract]) OR "cognitive declines"[Title/Abstract])) OR ("cognitive defect"[Title/Abstract]) OR "cognitive defects"[Title/Abstract])) OR ("cognitive deficit"[Title/Abstract]) OR "cognitive deficits"[Title/Abstract])) OR ("cognitive deficiencies"[Title/Abstract]) OR "cognitive deficiency"[Title/Abstract]) OR "cognitive deficient"[Title/Abstract])) OR ("cognitive degeneration"[Title/Abstract]) OR "cognitive delay"[Title/Abstract]) OR "cognitive delays"[Title/Abstract])) OR ("cognitive dementia"[Title/Abstract]) OR "cognitive deterioration"[Title/Abstract]) OR "cognitive deteriorations"[Title/Abstract])) OR "cognitive difficulties"[Title/Abstract]) OR "cognitive difficulty"[Title/Abstract]" OR ("cognitive disabilities"[Title/Abstract]) OR "cognitive disability"[Title/Abstract]") OR ("cognitive disadvantage"[Title/Abstract]) OR "cognitive disadvantages"[Title/Abstract])) OR ("cognitive disorder"[Title/Abstract]) OR "cognitive disorders"[Title/Abstract])) OR ("cognitive distortion"[Title/Abstract]) OR "cognitive distortions"[Title/Abstract])) OR ("cognitive disturbance"[Title/Abstract]) OR "cognitive disturbances"[Title/Abstract])) OR ("cognitive dysfunction"[Title/Abstract]) OR "cognitive dysfunctions"[Title/Abstract])) OR ("cognitive factor"[Title/Abstract]) OR "cognitive factors"[Title/Abstract])) OR ("cognitive handicap"[Title/Abstract]) OR "cognitive handicaps"[Title/Abstract])) OR ("cognitive illness"[Title/Abstract]) OR "cognitive illnesses"[Title/Abstract])) OR ("cognitive impaired"[Title/Abstract]) OR "cognitive impairing"[Title/Abstract])) OR ("cognitive impairment"[Title/Abstract]) OR "cognitive impairments"[Title/Abstract])) OR ("cognitive loss"[Title/Abstract]) OR "cognitive losses"[Title/Abstract])) OR ("cognitive perturbations"[Title/Abstract]) OR "cognitive problem"[Title/Abstract]) OR "cognitive problems"[Title/Abstract])) OR ("cognitive retardation"[Title/Abstract]) OR "cognitive symptom"[Title/Abstract]) OR "cognitive symptoms"[Title/Abstract])) OR ("cognitive restriction"[Title/Abstract]) OR "cognitive restrictions"[Title/Abstract])) OR ("cognitive troubles"[Title/Abstract] OR "cognitive vulnerabilities"[Title/Abstract] OR "cognitive vulnerability"[Title/Abstract])) OR ("cognitively impaired"[Title/Abstract]) OR ((("dementia"[Title/Abstract]) OR ("dementias"[Title/Abstract])) OR ("demential"[Title/Abstract])) OR ("demence"[Title/Abstract]) OR "alzheimer disease"[Title/Abstract] OR "alzheimer diseases"[Title/Abstract] OR "cognitive dysfunction"[MeSH Major Topic] OR "dementia"[MeSH Major Topic] | 248526 |
| #3 | #1 AND #2 | 1649 |
|  | |  |
