# Supplementary material for: Cognitive Impairment in Older Adults With Concurrent Hearing and Vision Impairment: A Systematic Scoping Review Protocol
Source: Front Psychiatry. 2021 Jul 19;12:661560. doi: 10.3389/fpsyt.2021.661560 (PMC8326366; doi:10.3389/fpsyt.2021.661560)
Supplement: Supplementary file 2 [file Data_Sheet_2.docx]

**Appendix II: Data extraction template**
